# Physical and Physiological Demands of Amateur Portuguese Field and Assistant Football Referees

**DOI:** 10.3390/sports12050133

**Published:** 2024-05-14

**Authors:** Ricardo Gomes, Rodrigo Mendes, Amaro Ferreira, Rui Mendes, Gonçalo Dias, Fernando Martins

**Affiliations:** 1 Applied Research Unit in Sport Sciences, Coimbra Education School, 3030-329 Coimbra, Portugal; rodsimendes@gmail.com (R.M.); rmendes@esec.pt (R.M.); goncalodias1976@sapo.pt (G.D.); fmlmartins@esec.pt (F.M.); 2 Polytechnic Institute of Coimbra, Coimbra Education School, 3030-329 Coimbra, Portugal; afc.amaroferreira@hotmail.com; 3 SPRINT Sport Physical Activity and Health Research & Innovation Center, 3030-329 Coimbra, Portugal; 4 Instituto de Telecomunicações (IT), Delegação da Covilhã, 6201-001 Covilhã, Portugal; 5 Research Unit for Sport and Physical Activity (CIDAF), Faculty of Sport Sciences and Physical Education, University of Coimbra, 3040-256 Coimbra, Portugal; 6 Football Association of Coimbra, 3030-330 Coimbra, Portugal; 7 InED—Centre for Research and Innovation in Education, Polytechnic Institute of Coimbra, 3030-329 Coimbra, Portugal

**Keywords:** GPS, football, match officials, match demands, external load

## Abstract

Referees are crucial elements in football, and they must meet the physical and physiological demands each match poses to them. The aim is to analyse the physical and physiological demands of amateur referees in games at the regional level (4th division), examining the differences between the first and second halves and between assistant (age: 25.10 ± 4.97) and main referees (age: 25.65 ± 5.12). A total of 29 matches were analysed with GPS devices, and internal and external load metrics were analysed. Overall, main referees, due to their central role in game management, showed higher levels of physical and physiological load than assistant referees, with more high-intensity activities, greater distance covered and higher heart rate. The results also revealed that there were no differences between the halves for total distance covered for either the main or assistant referees. However, the main referees covered a greater distance in high-intensity running during the first half (*p* = 0.05; d = 0.389). These findings emphasise the importance of tailored training protocols to enhance performance and reduce fatigue-related errors, highlighting the significance of endurance, high-intensity running ability, and strategies to manage transient fatigue in referee preparation.

## 1. Introduction

Main referees and assistant referees play pivotal roles in football matches and are responsible for interpreting and judging various occurrences on the field. Like players, referees must have adequate levels of physical fitness to effectively fulfil their duties during the game [1,2,3,4]. Previous studies [4,5,6] have examined the development of physical capacities and their impact on the performance of football referees, indicating that higher levels of cardiorespiratory capacity lead to improved referee performance.

A recent systematic review has found that factors such as age are not good discriminators of performance for referees [4], as older referees kept the same distance to the ball despite covering a shorter distance. Experience may play an important role in making older referees more efficient in their movement, eventually compensating for fewer intense actions and a shorter distance covered with better positioning.

The specific characteristics of and roles played by each type of referee in the game reveals the need to investigate the physical and physiological requirements of main referees and assistant referees, considering the diverse functions and actions of these officials. Studies have reported that both main referees and assistant referees cover substantial distances during international soccer matches, with referees covering more distance overall [6,7,8,9]. It has been observed, however, that main referees show higher values in terms of the total distance covered, average heart rate, and maximum speed compared to assistant referees [6,9,10,11,12], revealing the different needs and demands each official present during matches. Additionally, both groups experience high-intensity activity periods during matches [8]. A direct recommendation of these findings is that field referees should focus on training aimed at developing aerobic capacity and high-intensity running, while assistant referees should emphasise training for repeated short accelerations [9]. 

The demands placed on referees and assistant referees vary between different stages of the game. There is research indicating that the physical and physiological performance of referee teams tend to decline from the first to the second half, with an increase in intensity peaks [13,14,15]. Thus, in the first half, both groups cover more distance and experience more high-intensity activity periods compared to the second half, while assistant referees exhibit higher heart rates during the first half [8]. In addition, fluctuations in accelerations and high-intensity running have been observed between the first and second halves of matches, with a reduction in the assistant referees’ acceleration towards the end of each half [9]. This reduction in match performance has been attributed to a slower tempo of play [16], pointing to a relationship between the referees’ and players’ match load.

It has also been noted that the referees’ performance changes throughout the match, with lower values observed in the second half and transient fatigue occurring after peak 5 min periods [13,15]. On the other hand, neuromuscular fatigue may contribute to decreased performance, particularly in the last 15 min of the game [13,15]. Finally, soccer match officiating affects the physiological and physical performance measures of referees and assistant referees, resulting in decreased jump height, increased heart rate, and elevated blood lactate levels [7,15].

Match quality plays an important role in the referee’s physiological and physical responses. Referees tend to walk for greater total distances when officiating top-level matches, when compared with mixed matches [17]. Additionally, a recent study [1] found that the context where the matches are played also influence the referees’ performance. While elite referees cover an average of 10.45 km per match, differences were found when comparing European referees with their South American homologues (11.18 km vs. 9.32 km). However, these values are slightly lower than Barbero-Álvarez and colleagues’ research, where they reported values around 10.138 km [10]. 

A study has also compared the differences in match activity between female and male referees [18], finding that male referees have significantly higher intensity activity during matches. In Martin-Sanchez and colleagues’ study, male referees judged in male competitions, and female referees only participated in matches played by females, pointing that the physical preparation of referees should consider gender differences in match demands, ultimately leading to better performance. 

Research has focused on elite or sub-elite referees, with little information being available in lower category referees. Of the few studies analysed, Castillo-Rodríguez and colleagues analysed the differences between Spanish national and non-national referees [19], in which they found that non-national referees cover a shorter distance during matches. Accordingly, the average heart rate is also lower for the non-national referees. Another study has compared the referees of three different competitive levels in the Brazilian championships [12], confirming differences in match intensity and heart rate, both for main and assistant referees of different competitive levels. This reinforces the need to analyse the referees’ physical and physiological loads adapted to each competitive and social context, as this information is determinant to plan training strategies according to the specific needs of referees.

To our knowledge, very few studies have analysed Portuguese referees [1,14,15]. Within the Portuguese context, authors have centred their analysis solely on elite referees [15]. Hence, to bridge the gap in the current literature and characterise the match demands of amateur Portuguese referees, the aim is to analyse the physical and physiological demands of amateur referees in games at the regional level (4th division), examining differences between the first and second halves and between assistant and main referees. 

## 2. Materials and Methods

### 2.1. Participants

A total of 29 games were analysed, and data were collected from 17 referees (age: 25.65 ± 5.12; BMI: 24.11 ± 4.83) and 36 assistant referees (AR) (age: 25.10 ± 4.97; BMI: 23.88 ± 4.37). Data collection from one referee’s match resulted in an error, so total data analysed refers to 28 matches for the referees and 29 matches for the AR, resulting in a total data of 58 AR. The games were randomly selected from the highest amateur division of Portugal’s centre region.

### 2.2. Instruments and Procedures

**Figure 1:**
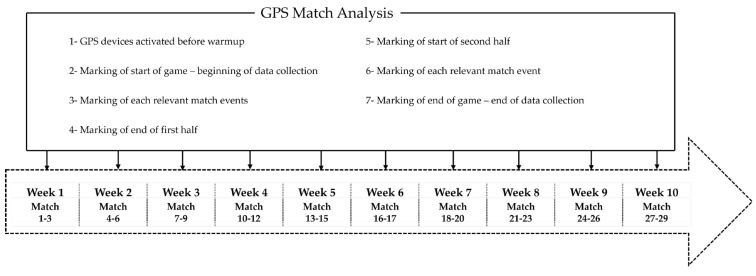
Visual representation of the timeline of the data collection procedure.

After approval from the regional referee council, the matches were selected randomly. Each week, 3 games were selected to analyse, in 3 different fields. The selected games occurred from October until December 2023, starting every Sunday at 15:00 (Figure 1). The weather conditions in which the games took place were not taken into consideration, although they were registered. A total of 3 games were played under rainy conditions, in the same week, and another group of 3 games happened under windy weather, again in the same week. The remaining games occurred under normal (sunny or cloudy) weather conditions. 

Data were collected using a GPS tracking system (WIMU Pro, Realtrack Systems, Almeria, Spain). These devices were placed on a sports vest in a way that did not interfere with any type of movement, these devices being commonly used in the sports world, validated for team sports [20] and used in related studies with referees [2,19,21,22]. Heart rate data were collected using a heart rate monitor, included in the GPS devices, using a Garmin heartrate strap. The devices were placed on the referees before the warm-up and were only removed at the end of the game. Data were selected to include solely the active sections of the game, with any interruptions such as assisting an injured athlete, breaks, substitutions, goal celebrations, and free-kick and penalty preparation being excluded from the analysis. The variables analysed were selected according to the literature [15,17,19]. Total distance (TD) was considered the total movement, in metres, of the referees during the match, after having excluded the interruptions. High-intensity running (HIR) is the distance covered by the referee at velocities over 13 km/h, and high-speed running (HSR) is the distance covered at velocities over 21 km/h. Accelerations (ACC) and decelerations (DECC) are the distances covered, in metres, while accelerating or decelerating above the threshold of 2 m/s^−2^. Average velocity was also analysed. 

### 2.3. Statistical Analysis

Comparisons between the referees’ first and second half, and total match load metrics were made using the dependent t test, after checking the normality and homogeneity assumptions [23]. This procedure was repeated for the assistant referees. Next, independent *t*-test was used to compare the global match load between referees and assistant referees, after checking the normality assumption [23]. For both the cases, Cohen’s *d* (*d*) effect size was estimated and interpreted using the follow criteria: no effect (ES < 0.2), small effect (0.2 ≤ ES < 0.5), large effect (0.5 ≤ ES < 0.8), and very large effect (ES ≥ 0.8) [24]. Statistical analysis was conducted using IBM SPSS Statistics (Version 28.0) software for Windows, and a statistical significance of 5% (*p* < 0.05) was defined.

### 2.4. Ethical Clearance

This investigation was performed according to the Scientific committee of the Polytechnic of Coimbra Coimbra Education School ethical regulations, and the project was approved and authorised by the Regional Referee Council of the Football Association. Informed consent was obtained from all participants, in accordance with the Declaration of Helsinki.

## 3. Results

A total of 29 matches were analysed, from which data from 28 main referees and 58 assistant referees were collected. Table 1 compares the differences between the match demands of the first and second half for the main referees. Overall, referees showed lower values in all variables analysed, except for acceleration (in metres).

Despite no differences in the total distance covered, referees were significantly slower in the second half (*p* < 0.001; *d* = 1.056; very large effect size). Referees also covered a shorter distance in high-intensity running (*p* = 0.05; *d* = 0.389; small effect size) during the second half. Significant differences in the average heart rate were also found. The referees also showed a lower maximum HR (*p* = 0.035; *d* = 0.419; small effect size), average HR (*p* = 0.003; *d* = 0.607; large effect size), and average %heart rate in the second half (*p* = 0.006; *d* = 0.615; large effect size).

A similar pattern was shown by the assistant referees, where their average velocity was significantly slower in the second half (*p* = 0.007; *d* = 0.369; small effect size), and the average %heart rate also being lower in the second half (*p* = 0.011; *d* = 0.347; small effect size). 

Table 2 presents the differences between the first and second half of the matches for the assistant referees.

Table 3 presents the differences between referees and assistant referees in the whole match. Statistically significant differences were found in all variables analysed.

## 4. Discussion

The comparison of match load demands between field and assistant football referees revealed interesting results. The games analysed were always played on Sunday afternoons, which is a common standard at this competitive level. However, referees usually must act in more matches per weekend, being common for them to make 2 to 3 games at the grassroots level before them showing up for the game. This heavy loading may have important consequences both in terms of performance and injury prevention. 

Considering the above, an average total distance covered per match of 9.39 km, at the 4th division competitive level, was not expected. A similar study compared national and non-national Spanish referees, and they found that non-national-level referees covered a total distance of about 5.5 km [19], which was a very low value. However, regional-level Brazilian referees cover about 10.8 km per match [12], a value that is close the values registered in top-tier referees in other European countries [1,2,8,13,19], and even within the Portuguese context, where a study that followed all Portuguese first league referees during one season found that the total distance covered was of 9.3 km [15].

One interpretation of these findings is that, in terms of total distance covered, match demands may not differ significantly between different competition levels, whereas the intensity at which these movements happen may be the key differentiating factor between competitive levels. Within the Portuguese context, elite referees cover about 2.5 km of HIR and 436 m of sprinting [15], which is significantly more than what was observed in the present study. At a regional level, comparing the results obtained with the ones found in the literature, referees run slower, produce fewer accelerations, and show a lower average velocity than their higher-level counterparts [2,15,19]. However, it must be noted that, as stated above, regional-level Portuguese referees usually perform after having refereed one, or even more grassroots-level games the day before or even on the same day of the match analysed. This match congestion may be another explanation to fewer high-intensity activities due to eventual signs of fatigue, as observed in top-level referees [25]. This interpretation is coherent with the fact that the reduction in the number of high-intensity activities is more evident during the second halves, which is accompanied with a reduction in the heart rate values. This event is expected, as such a heavy load requires a reduction in terms of match intensity. 

These assumptions increase the need to establish research focused in comparing the demands placed by matches of different levels on the referee, but also how a top-level referee would behave in a regional-level context. The following question arises: is it the context, that is, the level of the match, that influences the intensity at which referees perform their movements and actions, or is the fitness level of the referee limiting their capability to move? Additionally, how does match congestion limit the referees’ capacity to perform at this level?

When comparing the metrics in each half, no significant differences were found with the exception of the high-intensity running, average velocity, and the heart rate variables. The finding of similar match load demands in each half is not quite in line with other studies, where differences were found for the total distance covered [12,15], high-intensity running [12], and accelerations and decelerations [2,15]. It was expected that referees would produce significantly fewer accelerations and decelerations, as in similar research, due to eventual fatigue. However, this was not observed. One interpretation is that regional-level referees are less capable of producing more intense actions due to a lack of physical fitness, which may impair their capacity during whole game. As a result, referees would keep a relatively steady number of these activities during both halves of the match, mainly due to some lack of capacity of achieving the high-intensity thresholds. If so, specific training should be prescribed at this level, to increase their capacity of reaching these high-intensity thresholds, therefore increasing their ability to sustain more high-intensity activities during the match. 

Regarding heart rate response, our results show some similarity with a study involving Spanish first and second division referees, where they found that second division referees had a lower average and mean heart rate in the second half [2]. This is expected, as fatigue may force the referees to act at lower intensity levels to be able to endure through the whole match. When comparing heart rate values from Martinez-Torremoncha’s study [2] with our results, we may find that the referees from our study presented higher values for the first half (85.90% vs. 83.83%), and lower values for the second half (81.91% vs. 82.12%), indicating that physical fitness and fatigue may be a key differentiating factor between these two categories. 

A similar pattern was observed when analysing assistant referees. Total distance covered is in line with the values observed in the literature, without differences between the first and second half [12,13]. In terms of high-intensity activities, no differences between the first and second half were found neither for HIR and HSR, nor for accelerations and decelerations, which is also in line with the current literature [12]. These results may lead to two interpretations. On the one hand, and as stated above for the main referees, we may infer that a lack of physical fitness is a limiting factor for the ability to perform more high-intensity activities. If this assumption is true, referees should undergo specific training to increase their fitness levels, as their lack of fitness may be limiting their capacity of accompanying the match. On the other hand, another interpretation may be that, at a regional level, the intensity imposed by the players during the matches may not be sufficiently demanding to require high-intensity efforts by the ARs. Considering this to be true, the match demands are adequate to the fitness level of the referees. 

Regarding heart rate response, no differences were also found between the first and second half. These results maintain the idea that two interpretations may be valid. Either the ARs are not physically capable of producing enough high-intensity efforts due to lack of fitness, and therefore the first half is not intense enough, or the intensity demands for ARs are not high, and match intensity may be relatively stable at this competitive level. It would be interesting to investigate the relations of match load demands for players and referees at this level. Similar studies with professional or high-level matches have produced conflicting results [17,26]. 

The observed differences between the main referee and assistant referees suggest variations in roles and responsibilities. Main referees handle game rule enforcement and decision making, requiring more dynamic movements. Assistant referees, focusing on offside decisions and sideline support, involve relatively static positioning. The differences found are in line with the current literature and accompany what is observed with top-level referees [7,8,9,10,11].

These role differences likely contribute to varying match load demands. Main referees may exhibit higher physical demands due to their central role in game management, compared to assistant referees. Our findings underscore the need to consider distinct demands associated with different refereeing roles. Tailored training protocols could enhance performance and reduce fatigue-related errors. 

The difference in sample size between the main referees’ group and the assistant referees’ group is explained because there are twice as many assistant referees than main referees in each game. Future studies should consider increasing the number of main referees to reduce this discrepancy.

Future research could explore additional factors influencing match load demands, providing a more comprehensive understanding of this phenomena. For example, it would be interesting to understand not only the influence of match level, or intensity, on the referees’ match load. Additionally, another topic worthy of studying would be the relationship between technical and judgmental errors and internal match load, to understand how fatigue level impacts the referee’s number and types errors. 

This research was made in an ecological context, with maximum task representativeness. A limitation in this work that may be pointed is the difficulties in controlling the conditions in which the games were played. In natural contexts such as this one, there are many factors that may influence how a referee performs, which cannot be controlled. This may be faced both as a limitation and an advantage. Despite losing some reproducibility due to the difficulty in matching the exact contextual variables in which the games were played, it is irrefutable that task representativeness is present.

Like Martinez-Torremoncha’s study [2], some contextual variables were also not controlled. However, the games analysed were in the early season, a moment where no decisions regarding table positioning were made and the referees may have been less likely to be influenced by the pressure made by players, coaches, or fans. 

The results found in the present study can be used to inform the training and preparation of referees, highlighting the importance of endurance, high-intensity running ability, and strategies to manage transient fatigue [7,8,9,15,26]. Training strategies should be specific and meet the different physical and physiological demands that both assistant referees and main referees face during the match.

## 5. Conclusions

The physical and physiological demands placed on main referees and assistant referees during football matches are significant and should not be overlooked. The aim of this work was to analyse the physical and physiological demands of regional level amateur referees during the match, examining differences between the first and second halves and between assistant and main referees. Amateur-level referees have fewer high-intensity activities than their high-level counterparts, despite the similar distance covered. At an amateur level, both assistant and main referees show a decrease in high-intensity activities, average velocity, and heart rate from the first to the second half of the game. Referees must be able to meet the match demands, and understanding these demands, monitoring performance changes throughout a match, and implementing appropriate training and recovery strategies are essential for referees to enhance their performance on the field. By addressing these factors, we can improve the training and preparation of referees, contributing to the overall quality of the game.

## Figures and Tables

**Table 1:** Differences between match halves of referees.

	Match	1st Half	2nd Half	t	*p*	*d*
TD (m)	9386.35 ± 160.72	4703.42 ± 84.63	4682.94 ± 92.13	0.277	0.784	0.052
HIR (m)	668.69 ± 25.39	345.65 ± 14.15	323.04 ± 13.51	2.056	0.05 *	0.389
HSR (m)	204.22 ± 21.03	104.22 ± 12.83	100.00 ± 12.39	0.303	0.764	0.057
ACC (m)	795.08 ± 40.56	383.84 ± 22.15	411.24 ± 21.34	1.740	0.093	0.329
DECC (m)	1185.7 ± 47.75	607.67 ± 30.94	578.03 ± 21.88	1.218	0.234	0.230
AV. Veloc (km/h)	5.78 ± 0.11	6.05 ± 0.12	5.53 ± 0.11	5.588	<0.001 *	1.056
Max HR (bpm)	181.68 ± 2.23	180.71 ± 2.44	176.46 ± 2.31	2.217	0.035 *	0.419
AV HR (bpm)	152.61 ± 2.69	156.39 ± 2.99	148.82 ± 2.89	3.210	0.003 *	0.607
AV %HR	83.91 ± 0.76	85.90 ± 0.74	81.91 ± 1.16	3.254	0.003 *	0.615

* Statistically significant differences; *p* < 0.05.

**Table 2:** Differences in match demands between halves for assistant referees.

	Match	1st Half	2nd Half	t	*p*	*d*
TD (m)	5263.29 ± 55.10	2592.67 ± 35.79	2661.75 ± 39.85	−1.29	0.199	0.172
HIR (m)	477.57 ± 14.24	239.89 ± 8.07	236.85 ± 7.83	0.360	0.720	0.048
HSR (m)	89.41 ± 8.84	47.23 ± 5.21	42.09 ± 5.17	1.047	0.300	0.139
ACC (m)	588.67 ± 21.42	303.55 ± 12.61	285.02 ± 11.68	1.476	0.146	0.195
DECC (m)	671.18 ± 26.68	335.07 ± 15.75	336.85 ± 12.98	−0.156	0.877	0.021
AV. Veloc (km/h)	3.24 ± 0.03	3.33 ± 0.05	3.15 ± 0.05	2.789	0.007 *	0.369
Max HR (bpm)	173.02 ± 1.76	170.06 ± 1.87	168.23 ± 1.81	1.492	0.141	0.198
AV HR (bpm)	136.19 ± 1.70	137.60 ± 1.83	134.57 ± 1.70	2.683	0.010 *	0.355
AV %HR	78.66 ± 0.46	79.54 ± 0.54	77.85 ± 0.58	2.623	0.011 *	0.347

* Statistically significant differences; *p* < 0.05.

**Table 3:** Differences in match demands between field and assistant referees.

	Main Refs	Assistant Refs	t	*p*	*d*
TD (m)	9386.35 ± 160.72	5263.29 ± 55.10	12.287	<0.001 *	1.632
HIR (m)	668.69 ± 25.39	477.57 ± 14.24	6.221	<0.001 *	0.827
HSR (m)	204.22 ± 21.03	89.41 ± 8.84	8.170	<0.001 *	1.085
ACC (m)	795.08 ± 40.56	588.67 ± 21.42	5.064	<0.001 *	0.673
DECC (m)	1185.7 ± 47.75	671.18 ± 26.68	9.890	<0.001 *	1.314
AV. Veloc (km/h)	5.78 ± 0.11	3.24 ± 0.03	42.452	<0.001 *	5.640
Max HR (bpm)	181.68 ± 2.23	173.02 ± 1.76	5.393	<0.001 *	0.691
AV HR (bpm)	152.61 ± 2.69	136.19 ± 1.70	8.468	<0.001 *	1.183
AV %HR	83.91 ± 0.76	78.66 ± 0.46	8.308	<0.001 *	1.190

* Statistically significant differences; *p* < 0.05.

## Data Availability

The data presented in this study are available on request by the corresponding author due to privacy restriction.
